# Inner ear therapeutics and the war on hearing loss: systemic barriers to success

**DOI:** 10.3389/fnins.2023.1169122

**Published:** 2023-06-22

**Authors:** Nicole T. Jiam, Steven D. Rauch

**Affiliations:** Department of Otolaryngology – Head and Neck Surgery, Harvard Medical School, Massachusetts Eye and Ear, Boston, MA, United States

**Keywords:** inner ear, therapeutics, hearing restoration, hearing loss, drug development

## Abstract

Despite over 20 years of effort in academic research centers, start-up companies, and established pharmaceutical companies, there are no FDA-approved inner ear therapeutics for treatment of sensorineural hearing loss. There are a number of systemic barriers to creation of this new field of inner ear therapeutics. These include insufficient understanding of the particularity of different causes of hearing loss at the cellular and molecular level, lack of diagnostics of adequate sensitivity and specificity to discern these differences *in vivo*, a tendency for start-up biotech/pharma companies to prioritize competition over collaboration, and a drug development ecosystem that is really in the “pre-competitive” phase and a lack of infrastructure to develop, validate, gain regulatory approval, and successfully market an inner ear therapeutic. These issues will be discussed in this perspective article and a proposed remedy in the form of an inner ear therapeutics “moon shot” will be offered.

## Introduction

Hearing loss is one of the most common treatable health conditions in the world. It is a problem with profound economic and social impact. The World Health Organization completed and released results of an extensive study of the problem in 2021 (Olusanya et al., 2019; Deafness and hearing loss [Internet], 2023).

The causes of hearing loss, the societal impact, and the potential remedies vary between the developed and developing world. Simple, relatively “low tech” interventions to detect and treat childhood ear infections, to monitor and protect against high noise exposure, and to screen for significant hearing loss for early intervention with medications and hearing aids are predicted to be dramatically successful across the world’s developing countries. Recognizing that cochlear implants do not restore natural hearing, cutting edge academic research institutions, and biotechnology and biopharmaceutical industries are seeking innovative remedies for genetic and degenerative conditions that have historically been considered irreversible. These include the most common modifiable hearing health risk of all, acoustic trauma, as well as age-related hearing loss. In addition, these companies are also taking aim at less common causes of hearing loss, including ototoxic drugs used to treat other conditions, such as cancer chemotherapy agents and some antibiotics, idiopathic sudden sensorineural hearing loss, and much rarer conditions of hereditary/genetic deafness. These cutting edge, innovative treatments are promising, both to prevent deafness and to reverse it. However, despite at least 20 years of research, only one drug has been approved, quite recently, by the U.S. Food and Drug Administration (FDA) for use in humans: sodium thiosulfate as an otoprotectant to reduce risk of cisplatin ototoxicity in pediatric cancer patients with non-metastatic solid tumors (Maibach et al., 2014; Freyer et al., 2017). And neither that drug nor any other is currently available for clinical use in humans. Whether one considers conventional small molecule drugs, biologics, or gene therapy, the most advanced academic and industry efforts are still only in pre-clinical stages or early phase clinical trials. And perhaps most disheartening, with the exception of the cisplatin otoprotectant described above, to-date every drug brought to Phase II clinical trial has failed. Nonetheless, hopes are high and approximately 20 companies worldwide are continuing to develop these treatments.

Living with hearing loss presents many challenges. Regardless of whether symptoms arise early in life or late, gradually or suddenly, communication impairment is a heavy burden for hearing impaired people and their families, friends, and co-workers. If you have ever taken an airline flight, forgotten your headphones, and tried to watch a movie on someone else’s screen, across the aisle and two rows in front of you, you might have some small sense of the struggle hearing impaired people experience all the time. The cognitive load of coping with hearing loss is well-known (Meister et al., 2016). When such patients are “on their game” – well-rested, fit, and focused – they can often compensate quite well for their deficits. However, fatigue, stress, distraction, and other sensory or mobility impairments can be overwhelming, leading to significant communication impairment (Schulte et al., 2020). Clinicians and those who live with or around hearing-impaired individuals know that one common adaptive strategy is denial. Other people with hearing loss react by doing more and more talking, often unconsciously, to dominate conversation so they are not left behind or left out. Hearing loss often leads to social isolation (Shukla et al., 2020). There is a growing body of research showing that hearing loss is associated with accelerated cognitive decline in the elderly (Ray et al., 2018; Slade et al., 2020; Jiang et al., 2022).

Great breakthroughs in hearing restoration will rest, at a minimum, upon convergence of three critical lines of research: (i) Identification of “druggable” basic pathophysiologic mechanisms/disease targets, (ii) development of drug or other therapeutic agent to deliver, and (iii) identification or development of delivery system to get the agent to the target. Experts will offer differing assessments of the most promising avenues of hearing restoration research. However, all experts, both scientific and clinical, agree that they want these efforts to succeed. The literature in this field is abundant, and growing daily. What follows is a perspective of several systemic factors contributing to the failure, to-date, of these efforts to bring new pharmacologic and biologic treatments to our patients (Table 1).

**Table 1:** Systematic barriers in inner ear therapeutic drug development.

Issue	Barriers	Solution
Hearing loss is being measured as though it is a monolith	Crude clinical diagnostics and audiometric outcomes	Development of new audiometric, biochemical, serologic, and imaging diagnostic instruments
Hearing loss is rarely due to a very “focal” lesion	Unclear whether hearing restoration requires the exact recapitulation of the auditory developmental process	Understanding normal inner ear development and the role hearing restoration experiments affect these intertwined processes
The inner ear drug development pathway is unclear and untrodden	No FDA-approved otorestorative drug in the market	Intimate understanding of the therapeutic ecosystem and a collaborative moon shot effort

FDA, United States Food and Drug Administration.

### It’s a war

In January, 1971, President Richard Nixon signed into law the US National Cancer Act of 1971, which called for commitment of $1.5 billion (equivalent to $10 billion today) over 3 years for cancer research. In January, 2022, an editorial in Nature reviewed the 50 year history of this “war on cancer” (Nature, 2022). Most notably, it highlights that the war is not yet won. The editorial goes on to discuss a number of contributing factors, one of which is the fact that, even back in 1971, viewing the “disease” of cancer as a monolith was naïve. A great Egyptian pyramid that looks monolithic from a distance, upon closer inspection, is actually constructed of innumerable blocks piled and interlocking, one with another. Likewise, cancer is comprised of innumerable interlocking parts. It may arise from almost any cell type and in any organ, and the pathways of these malignant processes can differ widely at the molecular level. And like any war, progress is made in many small steps, battle by battle. There is a direct analogy here to our “war on hearing loss.” Hearing loss is not a monolith. But to the naïve, and even amongst expert clinicians in this early part of the 21st century, we are woefully ignorant of nuance and detail of the essential pathophysiology of hearing loss at the cellular and molecular level. We are making great strides in this area but have far to go.

**Figure 1:**
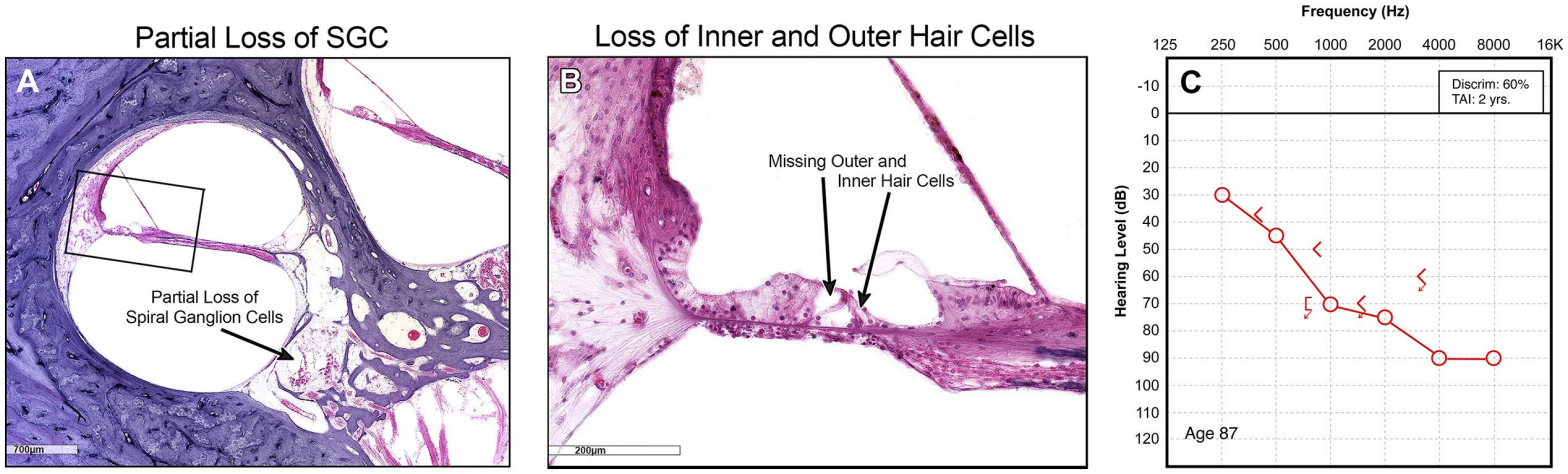
**(A)** Low power view shows partial depopulation of spiral ganglion neurons. **(B)** High power view of organ of Corti showing loss of inner and outer hair cells, and normal stria vascularis. **(C)** Corresponding audiogram.

**Figure 2:**
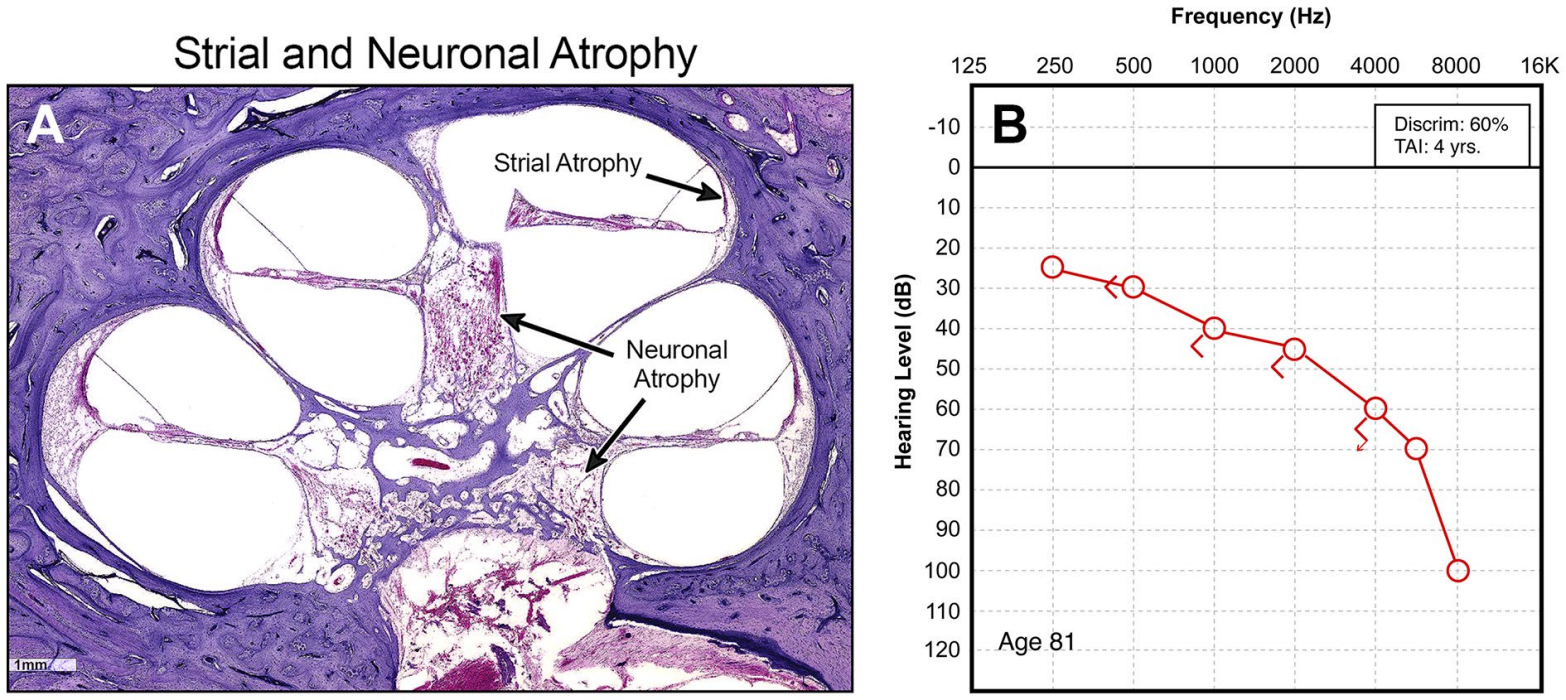
**(A)** Midmodiolar low power view of cochlea showing diffuse atrophy of spiral ganglion neurons and stria vascularis throughout the entire cochlea. **(B)** Corresponding audiogram.

**Figure 3:**
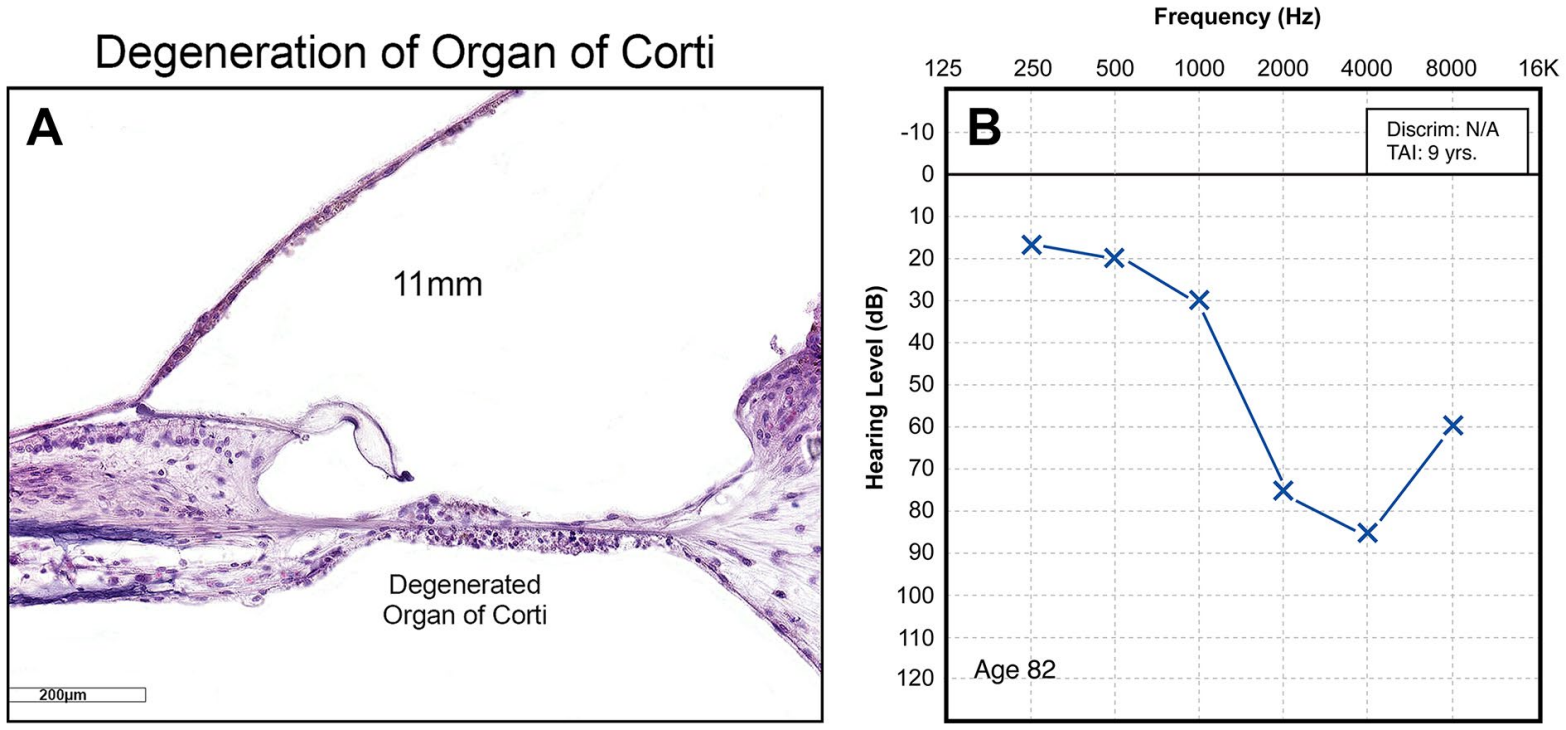
**(A)** High power view of organ of Corti showing obliteration of the entire organ, including hair cells, supporting cells, and pillar cells. **(B)** Corresponding audiogram.

The limits of our understanding of the “particular” nature of hearing loss at this granular level is also reflected in our clinical diagnostics. A behavioral audiogram is a blunt instrument. Pure-tone audiometry defines the psychophysical detection threshold for hearing at an array of frequencies believed to be clinically relevant when “modern” audiometers were invented 100 years ago. Figures 1–3 illustrate three examples of drastically different histopathology with only modest differences in pure tone auditory thresholds. Within the last 15 years, discovery of cochlear synaptopathy by Kujawa and Liberman (2009), and its subsequent confirmation across virtually all studied mammalian species, has brought to light an entirely new paradigm for understanding clinically relevant change in hearing threshold due to noise and/or age. Diagnostic audiometric methods in routine clinical use are insensitive to this pathology despite the fact that it may be the defining feature in the majority of cases of both noise-induced and age-related hearing loss.

Likewise, speech audiometry measuring word recognition score gives only the crudest assessment of hearing clarity. Emerging and slowly spreading use of speech-in-noise metrics represents early attempts to adopt clinical practices that are sensitive to cochlear synaptopathy and other nuances of sensorineural hearing loss (DiNino et al., 2022). Development of new diagnostic approaches, audiometric, as well as biochemical, serologic, via imaging, or other technologies as yet unknown, is a necessity. Only with these tools can we deconstruct the hearing loss monolith into its component parts so that scientists can develop targeted treatment strategies. It is entirely possible that some of the inner ear clinical trial failures that have occurred in the last 20 years were due to heterogeneity of the study population arising from inability to discern differences between hearing loss cases at the necessary level of granularity using current diagnostics. It is also possible that past failures may be due to audiometric outcome measures that were insufficiently sensitive or were off-target of the greatest treatment effect. The ingenuity, time, effort, and cost of developing, validating, and disseminating new diagnostics in clinical audiology are daunting. It is also mandatory if the field of inner ear therapeutics is to advance.

### It’s disaster relief

There is another good analogy for hearing restoration: Disaster relief. If you are a homeowner and a storm knocks off part of your chimney, it is a simple matter to call the mason and have them come over to do the repair. On the other hand, if your home has been devastated by a disaster, it matters little whether it was a flood, an earthquake, a tornado, or a fire. You will require an assortment of materials and workers in the building trades to reconstruct your home. In clinical otology and audiology, we may occasionally see a patient with a very “focal” lesion accounting for their hearing loss, for example, the monogenic COCH mutation of DFNA9 deafness (Robertson et al., 1998) or the many otoferlin mutations of DFNB9 deafness (Yasunaga et al., 1999). It is possible that repair or replacement of the defective gene could yield benefit for that patient. However, it appears that deafness of this type is the exception rather than the rule. Decades of postmortem study in temporal bones from patients who suffered age-related hearing loss, acoustic trauma, ototoxicity, other forms of chronic and progressive hearing loss, show devastation of the cochlea. Perhaps early in the evolution of this damage, it was confined to a single cell type, but as time passes, there is increasing and substantial collateral damage. Restoration of hearing in these ears will require restoration of some number of the 40+ different cell types in the cochlea, as well as control of both the sequencing and intensity of molecular signaling to control regenerative processes. It is well-known that normal inner ear development reflects elaborate cellular proliferation and differentiation, orchestrated by a complex array of neurotrophins, growth factors, intracellular, and molecular signaling, all occurring with precise sequencing, duration, and intensity (Elliott et al., 2021; Petitpré et al., 2022). Whether or not hearing restoration requires exact recapitulation of this developmental process is unknown. There may be some aspects of hearing restoration that are “self-assembling,” for example, new hair cells implanted into or otherwise grown in the organ of Corti might express signaling molecules that “call” neurites from the primary auditory neurons to come and create new synapses. Such mechanisms are, as yet, unknown. Basic science has been and continues to bring details of normal inner ear development to light, and to explore the ways these processes may occur in hearing restoration experiments, but we have a long way to go to understand this process and to manipulate it for clinical purposes. This is another domain where ingenuity and creativity, time, resources, and cost are daunting.

### It’s an ecosystem

If we step away from the ear for a moment and reflect on the topic of drug development in other fields, such as hypertension, depression, or chronic lung disease, there is a mature ecosystem of large established pharmaceutical companies using high throughput methodologies to screen candidate compounds, building and utilizing collaborations with smaller companies to outsource many aspects of drug development and screening, carry out preclinical and clinical trials, seek regulatory approval, and bring drugs to market. This ecosystem is regularly disrupted by new discoveries, most often from academic research centers, leading to entrepreneurial efforts to start a new company, develop the new drug, and for this start-up enterprise to either elbow its way into the ecosystem as a new player or be absorbed into it when taken over by one of the larger companies. Whether brought by a young company or legacy pharmaceutical company, when the new drug comes to market in an existing field, the pathway to regulatory approval is generally well-known. The efficacy metrics for the new drug and the comparator tolerability issues are well-known. When a new drug comes to market in an existing field, the target patient population is clearly identified. When a new drug comes to market in an existing field, the mechanisms to educate prescribing physicians and to educate patients are well-known and in place. When a new drug comes to market in an existing field, the ins and outs of seeking insurance reimbursement for that drug are also well-known and understood.

As noted in the introduction to this article, there are no FDA-approved inner ear therapeutics currently available for clinical use. There is no well-worn path to success for clinical trials design, selection of outcome measures, for regulatory approval, definition of target populations, dissemination of information to prescribing physicians and patients, or for negotiating reimbursement from health insurers. Developing “pipeline” drugs in other therapeutic areas often centers on increased potency compared to existing drugs. In the inner ear, where we are seeking to modulate developmental processes, increasing potency is more likely to cause catastrophic harm than improve control of complex events with critical sequencing and narrow concentration and timing parameters (Nyberg et al., 2019). These are nontrivial challenges. As noted above, we do not currently have diagnostics adequate to segregate our large population of hearing loss patients and identify the subset who might benefit from any particular therapeutic agent or intervention. The shortcomings of our diagnostics are not just in the area of patient identification. They are equally inadequate in defining treatment efficacy. Specific outcome measures for clinical trials are a necessity, both to judge efficacy of the drug or treatment as well as for seeking regulatory approval. And, even if we have the right outcome measure and even if we convince the FDA of the drug efficacy and obtain approval, that does not assure that an insurance company will pay for the drug. If the drug is lifesaving – e.g. a chemotherapeutic agent, drug for cardiovascular disease, etc. – reimbursement for the drug may be more likely. There has certainly been much publicity in recent months about gene therapy costing hundreds-of-thousands or even millions of dollars to treat a single patient (Castronuovo, 2023). Governments and insurance providers are struggling to develop reimbursement models. Restoring hearing will undeniably have impact on patients’ quality of life, but it is not lifesaving. If the primary benefit of an inner ear therapeutic is to improve speech recognition in noise, it may be difficult to convince an insurance company to pay many thousands of dollars so it is easier for someone to carry a conversation in a noisy restaurant.

One does not have to go the million dollar drugs to encounter reimbursement challenges. Consider a drug that must be administered in the otolaryngology office by intratympanic injection followed by an additional 30–60 min on the treatment table for the drug to be absorbed and/or to monitor for adverse effects. If the otolaryngologist is reimbursed $150 dollars for that treatment that occupies the treatment room for 1 hour, during which they might have seen four other patients at $75–$100 each, there is strong financial disincentive for adoption of the new treatment in clinical practice. Start-up companies have an existential need to achieve profitability. Publicly traded pharmaceutical companies are obviously committed to profitability for their shareholders. Current estimates are that the average cost of bringing the new drug to market is in the range of $1.3 billion (Wouters et al., 2020). In a totally new field with no comparator products or successful companies, business modeling is highly speculative. As a result, there is greater focus on short-term goals then on long-term success. Failure to appreciate the entirety of the therapeutics ecosystem may lead to long term failures even in those instances with early pre-clinical or clinical trials success.

### It’s a team effort

Whichever of the above analogies, warfare, disaster relief, or ecology, is most apt, they all share one critical feature: teamwork. Different units or branches of service in a military campaign seek to coordinate their efforts and pool their resources. Tradespersons on a construction project are not in competition. The plumber is not trying to out-do the electrician. In ecology, it is the complexities of interdependence that dictate success or failure. Serial entrepreneurs in the field of drug development who come into this new arena of inner ear therapeutics are likely to have finely honed competitive instincts and skills from their experiences in established fields, such as psychiatry or cardiovascular disease. Those are exactly the wrong instincts in this new field of inner ear therapeutics, which has yet to see any success. All the participants in the inner ear therapeutic space, the academic scientists, the entrepreneurs and start-up companies, the pharmaceutical industry giants, the clinicians in otology and audiology, are all in the same boat. If one company has a neurotrophin product and another company has a growth factor product and another company has a signaling molecule and another company grows hair cells, no one of them will succeed unless they all succeed. The infrastructure for successfully bringing an inner ear therapeutic agent to the patients who need it has not yet been built. We do not have the basic science understanding and control of normal developmental processes and pathologic processes leading to hearing loss. We do not have the diagnostics that are sufficiently granular and sufficiently sensitive to discern which patient has which problem. Likewise, we do not have the diagnostics to determine treatment efficacy. There is only limited success with regulatory approval to bring inner ear therapeutics into clinical trials and only one drug with primary inner ear mechanism of action (an otoprotectant, not an “otorestorative”) that has gained FDA approval. None have been brought to market. Thus, there is no experience with the physician and consumer education, distribution, or financing of such a treatment.

We are in the “pre-competitive” phase of inner ear therapeutics development. As noted above, meeting all the needs to build an infrastructure – the basic science, the diagnostics, the regulatory approval pathways, the distribution and marketing, and the financing – will require extraordinary ingenuity and creativity, time, human and other resources, and money. There is no single academic site or group of academic sites able to accomplish this. Government agencies such as NIH and DoD will play important roles in certain stages of the science but our government is not in the business of financing product development. There is no single start-up company or group of start-up companies with the financial resources and long-term security to enable this work. Though it may not have been done before, except perhaps in the very earliest days of the war on cancer, the best chance of creating a successful field of inner ear therapeutics would be for established pharmaceutical industry giants to set aside their competitive instincts and come together in a collaborative “moon shot” effort.

In 2021–22, Pfizer reported annual profits of approximately $66 billion, AstraZeneca $45 billion, Merck $41.5 billion, and Johnson & Johnson $63.8 billion. If each these companies pledged $1 billion annually for 10 years, and a consortium of experts from academe and industry was assembled to review applications and oversee disbursement of these funds, we would have our moon shot. We could expect to see rapid advancement in each of the domains necessary to establish an infrastructure for the field of inner ear therapeutics. By the end of this 10 year effort, there would be an expectation of transitioning to the competitive environment familiar in other fields.

Is it worth it? Our patients would certainly tell you that it is. Clinicians will agree. But there are many thorny legal, financial, and other logistical reasons this has not been done before. It is an undeniable fact that the original moon shot that put Americans on the moon in the 1960s and 70s gave rise to many advances in science and technology that were not the primary target of the project yet paid off again and again over the years. There is every reason to believe that similar collateral benefits would accrue from an inner ear therapeutics moon shot. Obviously, the major pharmaceutical companies who enable this project would need assurance that they, as well as the public and participants in the programs, would share in those downstream benefits. It will be challenging to design, negotiate, and execute such a program. It will never happen without tireless effort by hearing loss patients and their advocates fighting to see it done. It will require deft leadership, ideally by an individual recruited from outside this field but with keen scientific, financial, business, political, and interpersonal skills. It will be a monumental challenge. However, without an inner ear therapeutics moon shot program, we are likely to see many more years of failure and frustration. There is an African proverb that captures the essence of this undertaking: “If you want to go fast, go alone. If you want to go far, go together.”

## Author contributions

All authors listed have made a substantial, direct, and intellectual contribution to the work and approved it for publication.

## Conflict of interest

SA is the clinical advisory board member, Frequency Therapeutics, Inc., and the consultant, Spiral Therapeutics, Inc.

The remaining author declares that the research was conducted in the absence of any commercial or financial relationships that could be construed as a potential conflict of interest.

## Publisher’s note

All claims expressed in this article are solely those of the authors and do not necessarily represent those of their affiliated organizations, or those of the publisher, the editors and the reviewers. Any product that may be evaluated in this article, or claim that may be made by its manufacturer, is not guaranteed or endorsed by the publisher.
